# Review article: BK virus in systemic lupus erythematosus

**DOI:** 10.1186/s12969-015-0033-9

**Published:** 2015-08-21

**Authors:** Nirupama Gupta, Robert M. Lawrence, Cuong Nguyen, Renee F. Modica

**Affiliations:** Division of Nephrology, Department of Pediatrics, College of Medicine, University of Florida, Gainesville, FL 32610 USA; Division of Immunology, Rheumatology and Infectious Diseases, Department of Pediatrics, College of Medicine, University of Florida, Gainesville, FL 32610 USA; Department of Infectious Diseases and Pathology, College of Veterinary Medicine, University of Florida, Gainesville, FL 32610 USA

**Keywords:** BK virus, SLE, Immunosuppression

## Abstract

BK virus (BKV) is a human polyomavirus with a seroprevalence of 60–80 % in the general population. In renal transplant patients, it is known to cause renal failure, ureteric stenosis and hemorrhagic cystitis. In bone marrow transplant patients, it is evident that BKV can also cause hemorrhagic cystitis along with BK virus nephropathy (BKVN) in the native kidneys, with subsequent renal failure. However, little is known about BVKN in non-transplanted immune-compromised patients, such as systemic lupus erythematosus (SLE) who may have underlying nephritis and have a compromised immune system due to therapy and/or systemic illness. Thus, this article will focus on the clinical aspects of BKV and its association in patients with SLE.

## Introduction

BK virus (BKV) is a ubiquitous polyoma virus that is often acquired during early childhood. It lies dormant in the genito-urinary tract, but can become reactivated in certain immunocompromised disease states. The microenvironment needed for BKV replication includes an interplay between the viral characteristics, host’s altered or impaired immune system, inflammation and/or intrinsic kidney damage. BKV replication is observed after renal transplantation, and can lead to loss of the renal allograft in half of the cases. BKV replication is also observed in other solid-organ transplant recipients, bone marrow transplant recipients, human immunodeficiency virus (HIV) patients, pregnant women, multiple sclerosis and other immunocompromised patients. The use of immunosuppressive drugs, including biologics, strongly impacts the host’s immune system which increases the risk for certain opportunistic bacterial, viral, and fungal infections. In some particular groups of non-transplanted immune-compromised patients, such as systemic lupus erythematosus (SLE) who have a higher degree of renal involvement and/or systemic illness, the impact of BK virus replication is not known. Thus, this review article will focus on the describing the clinical aspects of BKV and a review of the current literature on BKV and its association with SLE.

## Review

### BK virus

#### History

BK virus is a human polyomavirus belonging to the family Papovaviridae. The other member of the Papovaviridae family is the *Papillomavirus* genus. The polyoma genus was named after the murine polyoma virus that caused tumors in newborn mice [1]. Polyomaviruses are ubiquitous in nature and are species-specific, including humans (JC virus [JCV], BK virus), monkeys (simian virus 40 [SV40]), and mice (mouse polyoma virus) [2]. BKV was first isolated in 1971 from the urine of a Sudanese renal transplant patient with ureteric stenosis, whose initials were B.K [3]. However, it was not until 1995 that the first report of BK virus nephropathy (BKVN) in a renal transplant recipient was published.

#### Genome

The BKV is a small (~45 nm) icosahedral, non-enveloped double stranded DNA virus composed of 5000 base pairs [2, 4]. The virus contains several domains: an early region consisting of the replicative genes, large tumor antigen (T antigen) and small tumor antigens (t antigen); a non-coding control region (NCCR) adjacent to the early region contains transcription factors for the early and late genes; and a late region encodes the viral capsid proteins (VP1, VP2, VP3) [4, 5]. The BKV uses the host cell for replication and does not incorporate into the host genome. There are four serologic BKV subtypes (I, II, III, and IV), with predominance of type I in 70–80 %, followed by type IV in 10–20 % [6]. The BKV genome also shares 75 % homology to the JCV and 70 % homology to SV40 virus [4]. JCV is more likely acquired at 10–14 years of age, with higher incidence of JC viruria than BK viruria in the general population at similar age range [4], and only a minority of cases (35 %) appear to co-activate BKV and JCV simultaneously [7].

#### Epidemiology

The primary BKV infection often occurs around the age of 3–4 years old [8], and once it is acquired, the virus lies dormant in the renal tubular epithelial cells [9]. BKV remains dormant in approximately 50 % of native kidneys, localized to the renal medulla [7]. In children under 10 years of age, the seroprevalence is about 50 % [10]; and, by adulthood, this increases to about 60–80 % [11]. Asymptomatic viruria occurs in both healthy and immunocompromised patients [12], with occurrence of < 5 % in the healthy population and about 60 % in immunocompromised patients [10]. For example, immunocompromised HIV positive patients with lower CD4 cell counts are known to have a higher prevalence of BK viruria [13].

Although BK viruria in most cases is not associated with nephropathy or hemorrhagic cystitis, certain populations have an increased risk of having BK viruria progress to viremia and subsequently develop BKVN. BKVN is the histological evidence of BKV-mediated tubulo-interstitial inflammation, which may eventually lead to renal failure.

In renal transplant patients, approximately 80 % of renal transplant recipients develop BK viruria [5], and 5–10 % of those go on to develop BKVN within a year of transplant [14], with loss of allograft function in about 50 % of the cases. In bone marrow transplant recipients, BKV-associated hemorrhagic cystitis is usually seen two to three weeks after transplant in 5–60 % of bone marrow transplant (BMT) recipients [4, 15]. Based on several case reports, BKVN may also affect the native kidneys of lung [16], stem cell [17–23], cardiac [17, 24–27] and solitary pancreas transplant recipients.

BKV reactivation with asymptomatic viruria also occurs in non-transplant patients on immunosuppression. The prevalence of BK viruria was 22 and 55 % in adult patients with multiple sclerosis [28] who received Natalizumab (an α4-integrin monoclonal antibody (mAb)) and those with inflammatory bowel disease [29] some of whom received Infliximab (an anti-tumor necrosis factor alpha (TNFα) chimeric mAb) and Adalimumab (anti-TNFα human mAb), respectively. Children with idiopathic nephrotic syndrome who were treated with Rituximab (an anti-CD20 (B-cell) chimeric mAb), on 6-month follow-up were noted to have 63 and 36 % of BK viruria and viremia, respectively [30]. However, the true prevalence of BKV in non-transplant patients may still be underestimated because this virus is not routinely screened in this population.

#### Pathogenesis

The precise mechanism of BKV transmission has yet to be determined; however, various routes have been implicated, such as: fecal-oral, respiratory, and organ transplantation [5]. Once acquired, the mechanism of dormant virus reactivation in certain patients is also not well known. It is thought to be stimulated by multiple predisposing factors related to the host immunity, viral load, allogeneic immune response (graft rejection or anti-lymphocyte antibodies) and inflammation (tissue injury, leukocyte infiltration, and the release of pro-inflammatory cytokines). Pre-existing damage to the kidney might be a contributing factor to invasive virus replication [31]. BKV reactivation may lead to progressive infection and renal tubular epithelial cell lysis. If the host’s immune system cannot mount BKV-specific immunity due to immunosuppressive medications, then renal dysfunction may ensue. One would assume that damage ensued by SLE nephritis has the appropriate milieu for BKV replication, but not enough data is available to clearly state whether this is a direct risk factor for BKVN development.

### Clinical manifestations of BKV infection

#### Genitourinary involvement

BK virus has tropism for the uroepithelial cells of the genitourinary tract. It is associated with a variety of complications in immunocompromised hosts, including: hemorrhagic cystitis, BKVN, and ureteral stenosis. The clinical presentation of BKVN is nonspecific with varying degrees of renal failure without fever, leukocytosis, hematuria or proteinuria. Often times the progression to BKVN occurs without clinical signs and symptoms, except for a rising serum creatinine over a period of one week. BKVN is usually preceded by the virus presence in the urine and subsequently in the plasma. BKV infection and rising creatinine can also be confused with graft rejection or other complications in renal and bone marrow transplant recipients, which poses a complex issue regarding therapy and management. Ureteral stenosis can present with urinary obstruction and elevated serum creatinine levels without symptoms of pain or discomfort since the transplanted kidney is deinnervated. Hemorrhagic cystitis in bone marrow transplant patients usually develops within two months of transplant and is presented with hematuria, dysuria, urgency, frequency, or suprapubic pain. With severe bleeding and clot formation, complications of urinary tract obstruction and renal failure may occur [2].

#### Other organ involvement

A few case reports have commented on the association of BKV infection in other non-renal organs, such as the lungs, eyes, liver, brain and tonsils [2, 31–33]. Goudsmit et al. [33] found BK viral DNA in the tonsils of children with recurrent attacks of upper respiratory infection, and Sundsjford et al. [34] found it in 2 of 201 nasopharyngeal aspirate specimens; however, in both groups, no infectious particles were isolated. Interstitial pneumonitis has been reported in at least two acquired immune deficiency syndrome (AIDS) patients, one of whom progressed to acute respiratory distress syndrome and subsequent fatality [35]. Another patient with AIDS, who presented with subacute visual deterioration and neurologic symptoms, was found to have BKV associated bilateral multifocal retinitis, which was detected by standard polymerase chain reaction (PCR) technique [36]. One case report mentions association between BKV and transaminitis in patients who were excreting the virus in the urine; however, no liver pathology report confirming this association have been published [37]. BK virus detection in brain tissue by PCR has been demonstrated in 20 to 100 % of non-HIV immunosuppressed patients with PML due to JCV [32]. Brain tissue for positive BKV DNA using PCR has been reported in 2 of 13 HIV infected patients who had progressive multifocal leukoencephalopathy (PML) and 1 of 16 HIV-infected patients who did not have PML [38]. However, low viral copy loads that are undetectable by PCR may give false negative results.

Thus, these reports raise the question of BKV tissue tropism, and how it becomes localized to the genitourinary tract when other organs may be the primary site of infection. These secondary sites of possible BKV infections also raises an important question of BKV association in SLE, which is a multisystem autoimmune disorder that can also affect the kidneys, brain, eyes, liver, lungs and other organ systems. Since BKV is not routinely assessed in all SLE patients, there is limited data on the association of BKV and its involvement in these other systems.

### Diagnosis

Both qualitative and quantitative assessments for BKV are available for diagnosis and management. Since viruria often precedes viremia by several weeks, urine cytology was first utilized to detect the presence of epithelial cells containing BK viral inclusions which were called “decoy cells”. Decoy cells are a morphological marker for BKV replication. Detection of decoy cells has a positive predictive value (PPV) of 27 % to indicate BKVN and negative predictive value (NPV) of 100 % [39]. However, the presence of decoy cells does not discriminate between lower urinary tract versus a renal parenchymal infection.

A more specific test to quantify the BK viral load from the urine and blood is via PCR. If the urinary BK virus PCR is negative, then it is more sensitive for excluding BKV as a cause of disease. A positive serum BK virus PCR has a PPV of 50–85 % for BKVN and a NPV of 100 % [14, 39]. Based on the transplant literature, when the urine BKV DNA >1 × 10^7^ copies/ml and/or plasma BKV DNA > 1 × 10^4^ copies/ml, a diagnosis of “presumptive BKVN” is made, even in the absence of demonstrable BKV replication in renal biopsies [40]. However, there is no established cutoff value for the level of viruria and viremia that is associated with BKVN in non-renal transplant patients.

**Fig. 1:**
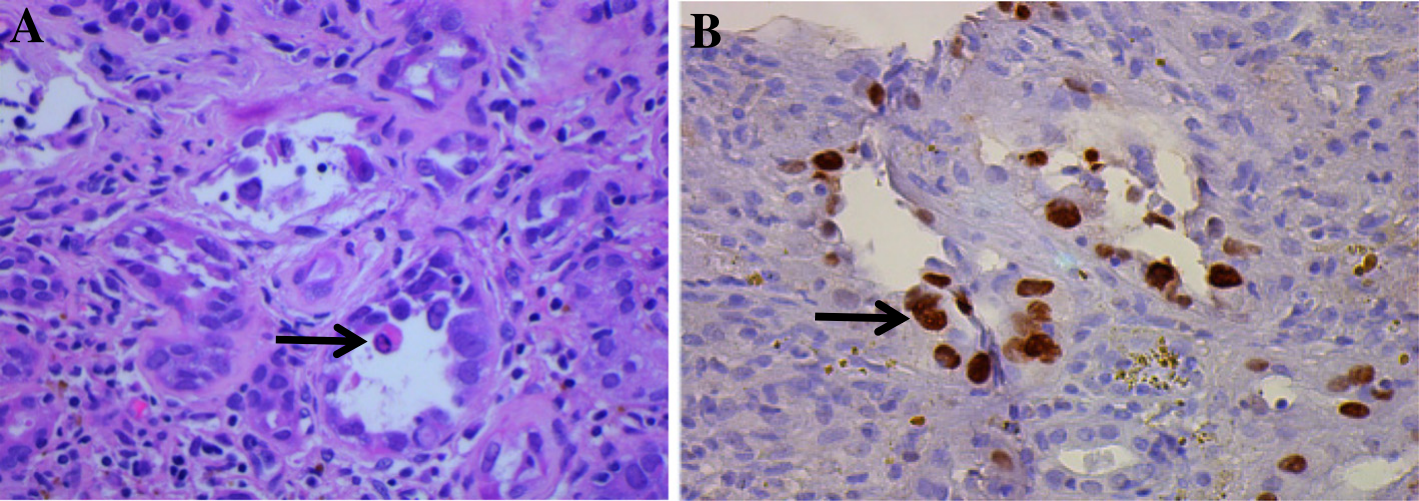
A native renal biopsy with multiple intranuclear BK viral inclusions (**a**) and positive staining for SV40 T antigen (**b**) in the tubular epithelial cells (identified by the arrows). There is also extensive interstitial fibrosis and tubular atrophy, along with prominence of inflammatory cells in the interstitium and tubular cells

A renal biopsy, to identify the typical intranuclear viral inclusion bodies in the renal tubular epithelial cells, remains the gold standard for BKVN diagnosis (see Fig. 1). However, even the renal biopsy can have false negatives of up to 30 % due to the focality of the BKV in the renal tissue [2, 41]. A positive immunohistochemistry staining for SV40 T-antigen is pathognomonic for BKVN (see Fig. 1). An electron microscopy shows polyomaviruses in the nuclei as crystalloid particles [42].

### BK virus and non-SLE immunocompromised patients

From the renal transplant literature, no single immunosuppressive agent definitively stands out as the culprit associated with BKV infection. The medications in transplant used most frequently include tacrolimus, mycophenolic acid (MMF), and cyclosporine, all of which inhibit cellular immunity thus affect T cell function and proliferation. Mengel et al. [43] showed that higher tacrolimus (FK) trough levels (>8 ng/ml) or MMF doses >1 g/day were associated with higher rates of BKV reactivation. Brennan [44] also reported higher BK viruria and viremia in the combination of FK-MMF (46 %) compared to the Cyclosporine-MMF (13 %) (*P* = 0.005). A retrospective study by Patel et al. [45] showed a higher incidence of developing viral and fungal infections in renal transplant patients who had received Rituximab compared to the no Rituximab group. BK viremia was present in 27 % versus 13 % in the Rituximab and no Rituximab groups, respectively (*p* = 0.011). It is plausible that Rituximab has long term effects on B-cell depletion and may decrease viral-specific IgG, and therefore, may hinder BK virus specific T-cell response [45]. Common medications used to treat some SLE patients may also include steroids, cyclophosphamide, MMF, Belimumab (anti-B lymphocyte stimulator) and Rituximab. MMF may be used to treat SLE nephritis, but may be given at higher doses than renal transplant patients (3000 mg/day compared to 2000 mg/day).

In 2009, Lonergan et al. [28] presented the first report of BKV reactivation in multiple sclerosis (MS) patients being treated with Natalizumab. This prospective study was conducted after MS patients were observed to have PML with Natalizumab. The group detected BK viruria in 8.3 % (3 of 36) prior to treatment and reactivation in 22.2 % (12 of 54). A longitudinal study by Delbue et al [30] reported 63 % (7 of 11) and 36 % (4 of 11) of BK viruria and viremia, respectively, at 6 month follow-up in children with idiopathic nephrotic syndrome who were treated with Rituximab,. BK viruria was detected in 3 of 11 patients before Rituximab, and only one had persistent viruria.

### BK virus in SLE

Systemic lupus erythematosus is an autoimmune disorder that can affect multiple organ systems, with up to 80 % of children presenting with renal involvement [46]. The disease manifests in childhood or adulthood, with higher female preponderance. The exact etiology of SLE is still unknown, but the pathogenesis of SLE includes the loss of tolerance to self-antigens and the formation of pathogenic antibodies directed against nuclear constituents (antinuclear antibodies (ANA), anti-double stranded DNA (dsDNA), anti-smith, ribonucleoprotein (RNP), SSA (anti-Ro), SSB (anti-La) and anti-histone) as well as complement mediated tissue destruction.

Since the 1980s, BKV infection was implicated in the pathogenesis of triggering SLE based on animal models. The development of anti-dsDNA appears to occur exclusively in SLE or Mixed Connective Tissue Disease whereas autoantibodies to histones are found in SLE, drug induced SLE and rheumatoid arthritis. Flaegstad et al. [47] demonstrated the production of antibodies to host polynucleosomes and histones in BKV inoculated rabbits. Moens et al. [48] demonstrated anti-dsDNA and anti-histone antibody production in the presence of a BKV T-antigen and host DNA complex in mice. Fenton et al. [49] demonstrated the affinity of nucleosome-T-antigen complexes for glomerular collagen IV and laminin by surface plasmon resonance in human renal biopsies from patients with SLE nephritis. The electron dense structures in the biopsies (along glomerular capillary membranes and within mesangial matrix) contained T-antigen, DNA and histones, which suggested that the extra-cellular chromatin may originate from polyomavirus infected cells in human kidneys. Hence, the release of these complexes may induce autoimmunity and be targeted by the induced pathogenic autoantibodies. Rekvig et al. [50] showed evidence for BKV reactivation in SLE patients by the simultaneous presence of BK viral DNA sequences in urine and anti-T-antigen antibodies in serum samples. The patients who had persistent viruria had higher titers for anti-T-antigen antibody and anti-DNA antibody titers.

However, the most current theory of pathogenesis is driven by the dysregulated clearance of apoptotic cells and development of autoantibodies via the process of “NETosis”. During NETosis, a specialized form of neutrophil cell death, the neutrophils extrude neutrophil extracellular traps (NETs). The NETs are composed of fibrillary networks composed of DNA, citrullinated histones, and granule peptides (neutrophil elastase, myeloperoxidase, and cathepsin G), which serve to entrap bacteria, viruses, fungi and parasites [51]. In SLE, there is an excessive activity of NETosis and impaired clearance of NETs, which results in an increased load of nuclear autoantigens and an increased antigenic and immunogenic potential [51]. Whether the presence of BK virus in the renal tissue of SLE nephritis patients implies that it is the cause of the renal symptoms or is a bystander is to still be determined. Hence, although causality of BKV and SLE has not been determined, an association may still exist.

There is significant correlation between the presence of BKV and the development of SLE. A first study by Taguchi et al. [52] first reported the isolation of BKV from the urine of a patient with SLE. They were able to demonstrate in two SLE patients the high serum antibody titers against BK virus (1:40 to 1:1280) by measuring cytopathic effect, the presence of urine decoy cells, and demonstrated the presence of BKV antigen by indirect immunofluorescent technique.

Since then, the prevalence of BKV viruria in SLE patients (see Table 1) has been reported at 16 % in SLE and 0 % in matched healthy controls. About 26 % of those patients continued to have persistent or recurrent BK viruria at 1–3 years follow-up [34]. Bendiksen et al. [53] showed that the BKV VP1 sequence was mainly of one strain type (MM) in both SLE patients and immunocompetent pregnant women in Norway. Different urine samples from the same patients over one year were predominantly stable, which suggested that reactivation of the viruses rather than recurrent or re-infections of patients with SLE was occurring. The mutation differences in VP1 sequences in this group were silent, with only a few missense mutations with amino acid substitutions. Moreover, the urine samples of the SLE patients did not possess JCV strains with a unique VP1 genotype, and only three individuals of 21 SLE patients had the presence of both BKV and JCV in the urine.

**Table 1:** Studies of BK Virus in SLE patients

Type of Study [Reference]	Number of SLE Patients	Subject Characteristics (Female/Male)	Method of Detection	Prevalence	
				BK viruria (SLE vs healthy controls)	BK viremia (SLE vs healthy controls)
Case series [52]	2	Adults	Cytopathic effect and Hemagglutinin in prototype BKV-infected cultures; decoy cells	Present	Not performed
Cross-sectional and prospective study [34]	44	Adults	PCR analysis of the NCCR region of BKV and JCV	16 % vs 0 % (BKV)	Not performed
		Study: (41/3)		11 % vs 21 % (JCV)	
		Control: (82/6)			
				Co-detection of BKV and JCV was not present	
Cross-sectional and prospective study [50]	20	Adults	PCR analysis of the NCCR region of BKV	43 % vs 4 %	Not performed
		Study: 20			
		Matched Control: 16^a^			
Cross-sectional study and prospective study [53]	5	Adults	Nested PCR analysis for BKV DNA	40 % vs 16 %	Not performed
		Study: (4/1)	Target: VP1		
		Control: (25/0)^b^	Primers: BKV-P1, BKV-P5, BKV-P2	Co-detection of BKV and JCV was present 4 % vs 0 %	
Cross-sectional study [54]	40	Adults^c^	Semi-quantitative BKV DNA PCR analysis (serum and urine)	32 % vs 17.2 %	15 % vs 13.8 %
		Study: (29/11)	Target BKV: pBK 385		
		Matched Control: 29^d^	Positive if BKV PCR >1000 copies/ml (viruria and viremia)		
				Simultaneous BK viruria and viremia 10 % vs 3.4 %	
Cross-sectional study [49]	7	Adults	Co-localization of anti-dsDNA and anti-T-antigen to glomeruli by immune electron microscopy	Not performed	Not performed
		Study: Renal biopsies			
		No control			
Cross-sectional study [55]	95	Children and Adults	Quantitative-real time BKV DNA PCR analysis	71.6 % vs 18.6 %	Not performed
		Study: 95	Target BKV: viral capsid protein (VP1)		
		Healthy Control: 32	Positive if BKV PCR >50,000 copies/ml (viruria)		
Cross-sectional study [56]	50	Children	Nested PCR analysis for BKV DNA	BKV: 32 %	Not performed
		Study: (41/9)	Primers: JC/BK_433F, JC/BK_4390F	JCV: 16 %	
		No control			
				Co-detection of BKV and JCV was not present	
Case Report [57]	1	Female Adolescent	Decoy cells in bright field microscopy	Present with 556 billion copies/ml	
			BKV tested by quantitative PCR		
Case Report [58]	1	Female Adult	Urine cytology with decoy cells	Not performed	Not performed
			Urine - qualitative PCR for BKV		
			Bone marrow – Positive SV40 stained cells		

*JCV* JC virus, *BKV* BK virus, *NCCR* Noncoding control region, *PCR* Polymerase chain reaction, *PYV* Polyoma virus

^a^4 subjects were rheumatoid arthritis patients

^b^Only those with SLE nephritis

^c^All control subjects were pregnant women

^d^11 subjects were health care workers

Colla et al. [54] reported BKV viremia in 15 % and BKV viruria in 32 % of adult SLE nephritis patients. The median number of genome copies/mL was 1.6 × 10^2^ in the blood and 1.6 × 10^4^ in the urine via PCR. Lu et al. [55] reported BKV viruria of 71.6 % vs 18.6 % in adult SLE and control patients, with a log of 4.74 vs 1.08 (urine BKV DNA copies/ml), respectively.

However, these studies did not find significant difference between BKV DNA positive and negative SLE patients in terms of SLE disease activity index (SLEDAI) score, presence of anti-dsDNA antibodies, CD4+/CD8+ ratio, and therapy. Interestingly, patients with positive BKV viruria exhibited a higher incidence of thrombocytopenia and higher complement C3 levels [55]. Rainthavorn et al. [56] reported that the prevalence of JCV and BKV reactivation was higher in pediatric SLE patients that in the general population. The study reported asymptomatic BKV viruria in 32 % of children with non-active SLE. This study also did not demonstrate a significant difference in the clinical characteristics (including age, hemoglobin, white blood cell count, platelet count, serum creatinine, complement 3, and frequency of pyuria or hematuria) among the BK viruria positive and negative groups. Additionally, Poloni et al. [57] reported a 17 year old female with lupus nephritis who was noted to have urinary decoy cells identified by bright field microscopy and BK viruria was confirmed by PCR (556 billion copies/ml). Umeda et al. [58] recently reported a 22 year old Japanese female with SLE who developed BKV-related hemorrhagic cystitis and hemophagocytic syndrome during her SLE flare treatment with immunosuppressive therapy, which consisted of methylprednisolone, calcineurin inhibitors (tacrolimus and cyclosporine), intravenous immunoglobulin and intravenous cyclophosphamide. The abdominal computed tomography showed thickened bladder, urine cytology showed decoy cells, and BK viruria was detected by PCR. The BK virus infection was also confirmed in the bone marrow by the presence of SV40-positive cells. With modification of the immunosuppressive therapy, BK viruria disappeared and both hemorrhagic cystitis and hemophagocytic syndrome resolved.

It is also important to note that the method of detection for BK viruria and/or viremia was different in all of the above-mentioned studies. It is possible that the sensitivity and specificity for BKV detection may have been impacted based on the region of BK viral genome amplification used for PCR and the type of PCR method employed (nested vs semi-quantitative versus real-time). Thus, with no standardized testing method for BK virus across centers, inter-laboratory and inter-study variability will limit comparisons and hamper identification of threshold cut off values. Also, only two of these studies had children in their inclusion criteria. The healthy matched control in Colla et al. study may have impacted the prevalence of BK infection since a significant number of control patients were healthcare workers.

### BK virus and immunosuppression in SLE

Bacterial, viral, fungal and parasitic infections are a major cause of morbidity and mortality in SLE patients. Respiratory (sinusitis, pharyngitis, bronchitis, pneumonia) and urinary tract infections are the most commonly involved sites [59]. One of three SLE patients die due to infection related complications; about 32 % SLE outpatients develop infections over two years; and sepsis can cause lupus nephritis to progress to end-stage renal disease [60].

The alteration of the innate and acquired immune systems in SLE patients increases the susceptibility to infection and this is exacerbated by the use of immunosuppressive medications (see Table 2); which affect both humoral and/or cellular immunity. Since an intact cellular immune response is required to clear viral infections, an impeded cellular immune response to elevated viral loads might contribute to the occurrence of BKVN [61]. Moreover, the current era of immunosuppressive therapy is now utilizing biologics at a higher frequency than in the past. Biologics may alter humoral immunity, cellular immunity and cytokine response, however, the long term effects of these drugs on the immune response is still unclear. Although long-term data is lacking, infection is still a major immediate concern.

**Table 2:** Infections associated with immune-modulatory drugs used in SLE

Immuno-modulatory Drugs [References]	Mechanism of Action	Immune Target	Viral Infections	Bacterial Infections	Fungal Infections
Glucocorticoids [60, 62, 64, 71]	Inhibition of NF-kB	Impact all immune cell types, nonspecific	CMV	*Mycobacteria*	Candida
			HSV	*Nocardia*	Cryptococcus
			VZV		PCP
			Measles		
			Kaposi’s sarcoma		
Cyclophosphamide [62, 64, 72]	DNA alkylating agent	Impact all immune cell types, nonspecific	CMV	*Mycobacteria*	PCP
			HSV	*S. epidermidis*	
			Viral hepatitis		
MMF [62, 64–66, 73]	Inhibition of IMPDH/de-novo purine synthesis inhibitor	B and T cells	CMV	(No specific organism identified)	Aspergillus
			HSV		Candida
			JC virus		Cryptococcosis
			VZV		Mucor
					PCP
Rituximab [45, 60, 67–70, 74]	Chimeric human-mouse IgG1 anti-CD20 monoclonal antibody	B cells	BK virus	*E.coli*	Aspergillus
			CMV	*Mycobacteria*	Candida
			EBV	*P. aeruginosa*	Nonaspergillus mould
			Enterovirus	*S. aures*	PCP
			HSV	*S. typhii*	Tinea corpis
			Hepatitis B & C	*Shigella*	
			Influneza A	*Streptococcus* spp.	
			JC virus, Parvovirus B19		
			RSV		
			VZV		
			West Nile virus		
Belimumab [75]	Human IgG1 antibody that binds to soluble B-lymphocyte stimulator (BLyS or BAFF)	B cells	CMV	*Acinetobacter*	Coccidomycosis
			Influenza	*Mycobacteria*	
				Pneumonia	
				UTI	
				Cellulitis	
Abatacept [69]	Fully human soluble fusion protein, Anti-CTLA4	Antigen presenting cells	HSV	*Mycobacteria*	Aspergillus
			VZV	Pneumonia	Candida
				Sepsis	
				Skin infections	
				URI	
Methotrexate [60]	Anti-folate synthesis inhibitor	B and T cells	CMV	*Listeria*	Aspergillus
			Hepatitis B virus	*Mycobacteria*	Histoplasma
			HSV	*Nocardia*	PCP
			JC virus		
			VZV		

*MMF* Mycophenolic acid, *VZV* Varicella zoster virus, *CMV* Cytomegalovirus, *HSV* Herpes simplex virus, *EBV* Ebstein-Barr virus, *VZV* Varicella zoster virus, *RSV* Respiratory syncytial virus, *UTI* Urinary tract infection, *PCP Pneumocystis carinii* pneumonia, *URI* Upper respiratory infection

In SLE patients, the increased risk that immunosuppressive agent(s) contribute to the risk of BKV infection or reactivations is not known. Steroids and cyclophosphamide are associated with a strong risk for infection [62], while antimalarials may have a protective role [63]. Use of biologic therapeutic agents that have been reported in SLE patients include: Rituximab, Belimumab, and Abatacept (anti-CTLA4 Ig). Rituximab, used for autoimmune cytopenias associated with SLE, and Belimumab block B cell activation. Abatacept, used in patients with arthritis and lupus, modulates co-stimulation of T-cells by antigen presenting cells. It is important to note that the patients in Colla et al. and Lu et al. studies had not received any biologics for therapy. The medications reported in their studies included: methylprednisolone, prednisone, cyclophosphamide, intravenous immunoglobulin (IVIG), azathioprine, cyclosporine, or MMF. Therefore, it is not known if biologics are more immunosuppressive and/or pose a higher risk for BKV reactivation in SLE patients. Currently, BKV infection has been reported in SLE patients on Rituximab. The association of BKV infection with other medications listed in Table 2 is yet to be revealed. Since routine use of these biologics in SLE has been more recent, the long-term infectious profile of these drugs is uncertain and requires monitoring. Furthermore biologics may be used concomitantly with steroids and more traditional immunosuppressive agents leading to multiple medications hampering the immune response by different mechanisms. It is difficult to ascertain to what degree of cellular or humoral impairment will lead to activation of BKV if multiple immune pathways are dysregulated simultaneously.

### Prognosis

BK virus nephropathy is an irreversible condition that can lead to renal failure. Since BKVN occurs in up to 10 % of renal transplant recipients, with half losing their allograft, screening for BK virus infection is part of routine care in a renal transplant patient. However, this is not the case for other non-renal transplant or immunosuppressed patients. Increase in serum creatinine as a marker for renal dysfunction may be too late in the disease process since the glomerular filtration rate has already decreased by half by that time. Thus, although a firm recommendation to obtain routine BKV screening in all SLE patients cannot be made from this review, clinicians should consider BKV screening as clinically indicated, especially in the setting of proteinuria, hematuria, and/or rise in serum creatinine. A simple test for BKV may change management and tailoring of therapies for such vulnerable patients. Further research is needed to determine the formal recommendations for BKV screening and monitoring in this patient population.

### Treatment

Once there is detection of BK viruria and viremia, the treatment strategy for BKV infection is not uniform and will need further investigation. Extrapolation from the renal transplant literature reveals that an acceptable treatment plan involves reducing immunosuppressive medications and monitoring serum creatinine and BKV load periodically. Ancillary therapies such as cidofovir [76], leflunomide [77, 78], quinolones [79, 80] and IVIG [81, 82] have been employed, but they have not proven to be more efficacious than screening and reduction of immunosuppressive therapy in renal transplant recipients (see Table 3). The reduction of immunosuppression in SLE patients in order to allow better host clearance of the virus could potentially have deleterious effects on their underling autoimmune disease and is therefore a limited option and requires further consideration.

**Table 3:** Treatment Options for BK virus infection from the renal transplant literature

Drug [Reference]	Mechanism of action	Dose	Adverse effect
Cidofovir [76]	Synthetic purine nucleotide analogue of cytosine viral DNA polymerase inhibitor	0.25 to 1.0 mg/kg at 1–3 weekly intervals without probenecid	Nephrotoxicity
Leflunomide [77, 78]	De novo pyrimidine synthesis inhibitor	Loading dose of 100 mg for 5 days, then maintenance dose of 20–60 mg/day	Hepatic dysfunction Anemia
		Target blood level of 50 to 100 ug/mL	
Fluoroquinolones [79, 80]	DNA gyrase inhibitor (interferes with T antigen helicase activity in BKV)	Levofloxacin 500 mg/day x 1 month	Tendinitis
		Ciprofloxacin 250 mg BID daily x 1 month	
IVIG [81, 82]	Direct neutralizing activity and other immune-modulatory effects	Variable doses of 0.2 to 2.0 g/kg:	Osmotic nephropathy
		1) 600 mg/kg every 4–6 weeks	Headache
		2) 2 g/kg over 5–6 days	Aseptic meningitis
			Thrombotic complication
			Hemolysis

## Conclusions

It is important to delineate the interaction between BKV, the altered immune system and the affected kidneys of SLE patients to fully understand how to effectively diagnose and manage BKV infection in these patients. This information may impact immunosuppression therapy choices, which may also affect the control of the autoimmune disease. At this time, more information is still needed about the prevalence and incidence of BKV infection in SLE, especially children, with and without nephritis. More studies are needed to establish the threshold cut-off values for significant BK viral load and risk for BKVN in non-transplant immunocompromised patients. Furthermore, randomized controlled trails and algorithms are needed to identify the best treatment strategies to treat at-risk children and adults who acquire BKV infection, especially in the non-renal transplant setting. In summary, by understanding the relationship between BKV and the altered immune system in SLE, we may be able to extrapolate this information to other at-risk populations to impact proper surveillance, diagnosis and treatment.

## Abbreviations

AIDS: Acquired immune deficiency syndrome
ANA: Antinuclear antibodies
BKV: BK virus
BKVN: BK virus nephropathy
DNA: Deoxyribonucleic acid
dsDNA: Anti-double stranded DNA
HIV: Human immunodeficiency virus
IVIG: Intravenous immunoglobulin
JCV: JC virus
mAb: Monoclonal antibody
MMF: Mycophenolic acid
MS: Multiple sclerosis
NCCR: Non-coding control region
NPV: Negative predictive value
PCR: Polymerase chain reaction
PML: Progressive multifocal encephalopathy
PPV: Positive predictive value
RNP: Ribonucleoprotein
SLE: Systemic lupus erythematosus
SLEDAI: SLE disease activity index
SSA: Anti-Ro
SSB: Anti-La
SV40: Simian virus 40
TNFα: Tumor necrosis factor alpha
VP1: Viral capsid protein 1
